# Potentiation of the Antimicrobial Effect of Oxytetracycline Combined with Cinnamon, Clove, Oregano, and Red Thyme Essential Oils against MDR *Salmonella enterica* Strains

**DOI:** 10.3390/ani14091347

**Published:** 2024-04-30

**Authors:** Belén Huerta Lorenzo, Ángela Galán-Relaño, Emilio Barba-Sánchez, Antonio Romero-Salmoral, Ana L. Solarte Portilla, Lidia Gómez-Gascón, Rafael J. Astorga Márquez

**Affiliations:** 1 Animal Health Department, Veterinary Faculty, University of Cordoba, 14014 Cordoba, Spain; sa2hulob@uco.es (B.H.L.); emilio.barba@hotmail.com (E.B.-S.); antromsal98@gmail.com (A.R.-S.); lulitasolarte@gmail.com (A.L.S.P.); v32gogal@uco.es (L.G.-G.); sa1asmar@uco.es (R.J.A.M.); 2 Zoonotic and Emerging Diseases (ENZOEM), University of Cordoba, 14014 Cordoba, Spain; 3 Mariana University, Calle 18 No. 34-104 Pasto (N), San Juan de Pasto 52001, Colombia

**Keywords:** antimicrobial resistance, MDR, essential oils (EOs), interaction, synergism

## Abstract

**Simple Summary:**

*Salmonella* spp. of both human and animal origin have a high resistance percentage to tetracyclines. Essential oils, including cinnamon, clove, oregano, and red thyme, have demonstrated bactericidal activity against this bacterium. However, in many cases, the minimum inhibitory concentration (MIC) exceeds the cytotoxicity limits. The aim of this study was to evaluate the in vitro effectiveness of combining oxytetracycline with essential oils against multidrug-resistant *Salmonella enterica* strains. The results indicated a positive interaction (synergy and additivity) between oxytetracycline and the four oils that were tested. This led to a reduction in the MIC of both the oils and the antibiotic. The reduction was between 2 and 4 times the initial value for the oils and between 2 and 1024 times for the antibiotic. The best results were achieved with the combination of oxytetracycline and cinnamon, which decreased the effective concentration of this antibiotic to below the sensitivity threshold. Although differences in response were observed depending on the bacterial strain, there was no antagonistic effect in any case. The study suggests that combining oxytetracycline with cinnamon oil may be an effective alternative for controlling tetracycline-resistant strains of *Salmonella*, although further studies would be advisable.

**Abstract:**

Tetracyclines have a high resistance percentage in *Salmonella* spp. of both human and animal origin. Essential oils, such as cinnamon (*Cinnamomum zeylanicum*), clove (*Eugenia caryophyllata*), oregano (*Origanum vulgare*), and red thyme (*Thymus zygis*), have shown bactericidal activity against this bacterium. However, in many cases, the minimum inhibitory concentration (MIC) exceeds the cytotoxicity limits. The objective of this study was to assess the in vitro efficacy of combining oxytetracycline with essential these oils against field multidrug-resistant (MDR) *Salmonella enterica* strains. The MIC of each product was determined using the broth microdilution method. The interaction was evaluated using the checkerboard method, by means of the fractional inhibitory concentration index (FIC_index_) determination. The results showed a positive interaction (synergy and additivity) between oxytetracycline and the four oils tested, resulting in a reduction in both products’ MICs by 2 to 4 times their initial value, in the case of oils, and by 2 to 1024 times in the case of the antibiotic. The combination of oxytetracycline and cinnamon achieved the best results (FIC_index_ 0.5), with a decrease in the antibiotic effective concentration to below the sensitivity threshold (MIC of the combined oxytetracycline 0.5 µg/mL). There was no antagonistic effect in any case, although differences in response were observed depending on the bacterial strain. The results of this study suggest that combining oxytetracycline with cinnamon oil could be an effective alternative for controlling tetracycline-resistant strains of *Salmonella*. However, its individual use should be further evaluated through in vitro susceptibility tests.

## 1. Introduction

*Salmonella* is one of the main bacteria responsible for foodborne illnesses, and it is considered a zoonotic agent with a significant impact on public health [1]. Traditionally, controlling this infection in humans and animals has relied on broad-spectrum antibiotic therapies. However, the irrational use or overuse of these drugs in veterinary and human medicine has led to the development of antibiotic resistance in pathogenic bacteria, including *Salmonella* [2,3]. As a consequence, in 2006, the EU banned the use of antimicrobials as growth promoters in animals. Despite these limitations, recent reports from the European Food Safety Authority (EFSA) and the European Centre for Disease Prevention and Control (ECDC) show high resistance of *Salmonella* spp. to ampicillin, sulfamethoxazole, and tetracyclines, with resistance rates for the latter reaching 26.4% in humans and 32.9% in animals [4]. Unfortunately, the rate at which antibiotic-resistant bacteria evolve is much faster than the rate at which new antibiotics are discovered [5]. Despite significant efforts, no new class of antibiotics has been discovered in the past 20 years. It is important to consider a practical approach to the use of currently available antibiotics, with a focus on inhibiting or reversing the development of resistance in pathogenic bacteria, given the lack of effective antibiotic alternatives [6].

In the last decade, research has focused on the possibility of combining traditional antibiotics with natural antimicrobials, such as essential oils, to increase or restore their effectiveness against MDR bacteria [7]. The studies conducted showed varying results depending on the bacterial species and products used. However, in general, they describe an increase in bacterial susceptibility [8,9,10,11].

Essential oils (EOs) are a complex mixture of between 20 and 60 chemical compounds, with two or three accounting for the majority (>70–80%) and the rest present in trace amounts. The proportion of each substance can vary depending on the season, geographical origin, botanical variety, plant genetics, or extraction method, resulting in different chemotypes [12]. The main components can be divided into two groups based on their biosynthetic origin: terpenoid hydrocarbons (terpenes and terpenoids) and aromatic and aliphatic compounds. Among the terpenes, the most important active principles are the monoterpenes and sesquiterpenes [13,14]. Most EOs are products Generally Recognized as Safe (GRAS) by the United States Food and Drug Administration (FDA) and are currently authorized for use in food as food additives [15]. Nevertheless, they can have a dose-dependent cytotoxic effect [16].

*In vitro* studies have shown that oregano (main active component: carvacrol), thyme (thymol), clove (eugenol), cinnamon (eugenol, cinnamaldehyde), and EOs are remarkably effective against Gram-negative bacteria [17,18]. The antimicrobial effect is due to the combined action of several mechanisms on different cell locations: (i) disruption and permeabilization of the cell membrane, (ii) aging of ATP and potassium/hydrogen ions, (iii) inhibition of enzymatic activity, among others. This also makes it difficult for bacterial resistance to develop [19,20,21]. Furthermore, previous studies have demonstrated that the combination of cinnamon and thyme has an additive effect against Gram-positive and Gram-negative bacteria (*Bacillus* spp., *Staphylococcus aureus*, *E. coli*, and *S.* Typhimurium) [22]. The combination of cinnamon, clove, oregano, and red thyme EOs with enrofloxacin, ceftiofur, and trimethoprim-sulfamethoxazole demonstrated a synergistic effect against multi-resistant strains of Salmonella enterica [11].

The aim of this study was to evaluate the in vitro antimicrobial potential of the combination of oxytetracycline (OT) with cinnamon, clove, oregano, and red thyme EOs against multidrug-resistant field strains of *Salmonella enterica*.

## 2. Materials and Methods

### 2.1. Bacterial Isolates

Five isolates of *Salmonella enterica* subspecies *enterica* that were multiresistant and not susceptible to OT (determined by antibiogram [23]) were used. These isolates were obtained from the Collection of Cultures of the Animal Health Department of the University of Cordoba and from the National Reference Laboratory for *Salmonella* and *Shigella* (Madrid, Spain). The reference strain of *Salmonella* Typhimurium ATCC14028 was included as a quality control. All strains were stored in frozen cryoballs at −20 °C (CRYOBANK™, London, UK) until use.

Table 1 provides information on the origin, serotyping, phage typing, and antimicrobial resistance profile of the isolates used in this study [11].

### 2.2. Antimicrobial Agents

OT hydrochloride VETRANAL from Sigma-Aldrich Laboratories (USA) was used for antimicrobial sensitivity analysis. The solution was prepared by diluting OT hydrochloride in sterile distilled water to obtain a concentration of 4096 μg/mL [23].

Cinnamon, oregano, clove, and red thyme EOs (purity ≥ 95%) were purchased from Aromium™ (Barcelona, Spain). The chemotype of each EO, determined by manufacturer using Gas-Chromatography analysis, is listed in Table 2. All the products were stored at room temperature in the dark prior to testing, following the manufacturer’s instructions.

### 2.3. Susceptibility Test of OT and EOs

Following the broth microdilution method [23], double serial dilutions of OT in sterile distilled water (2–2048 μg/mL) were prepared and challenged with an equal volume of bacterial inoculum of 10^6^ CFU/mL. For EOs, double serial dilutions (156.25–20,000 μg/mL) were prepared in Müller–Hinton broth supplemented with 0.15% agar (MHB) (Oxoid Ltd., Wade Road, Basingstoke, Hampshire, RG24 8PW, United Kingdom). The essay was conducted in triplicate with different inocula, including every time there was a positive control for plate counting (MHB with bacterial inoculum and no product), and a negative control (MHB without inoculum and no product). After incubation at 37 °C for 24 h, the individual minimum inhibitory concentration (MIC) was estimated as the lowest product concentration capable of inhibiting visible bacterial growth in the plate wells, determined by visual comparation with the positive and negative controls. The final value was taken as the median of the three assays.

### 2.4. Antimicrobial Interaction Test

The combined effect of OT with each EO was evaluated using the checkerboard method described by Si et al. [24]. A volume of 50 µL of each one of the eleven serial double dilutions of OT was tested against the same volume of the seven serial double dilutions of EOs (50 µL) in a 96-well microtiter plate. Thus, the dilutions ranged from 4× MIC_I_ to 0.0039× MIC_I_ for OT, and from 4× MIC_I_ to 0.062× MIC_I_ for Eos, as shown in the schedule below.



**Antimicrobial Dilution with Respect to MIC_I_ (µg/mL)**


4×2×1×0.5×0.25×0.125×0.0625×0.03125×0.0078125×0.0039×

C+









**Natural product dilution with respect to MIC_I_ (µg/mL)**4×










2×










1×










0.5×










0.25×










0.125×










0.0625×












Subsequently, 100 µL of bacterial inoculum at a concentration of 10^6^ CFU/mL was added to each well. Each well contained 100 μL of dilution and 100 μL of bacterial inoculum. As a consequence of this test, the MIC of each product was again determined. From this point forward, this MIC determined in the interaction test will be referred to as individual MIC (MIC_I_). The MIC_I_ of each product (OT: MIC_OT_ and EOs: MIC_EO_) against the tested inoculum was determined using the first row and first column of each plate. All tests were conducted in duplicate and included positive and negative growth controls. The plates were incubated at 37 °C for 24 h. As a result of combining the products, a new concept arises: the combined minimum inhibitory concentration (MIC_C_). This is defined as the concentration of the compound (OT or EO) necessary to inhibit the growth of the strain in the presence of the other compound.

The in vitro effect of each OT–EO combination was determined by calculation of the fractional inhibitory concentration (FIC) and fractional inhibitory concentration index (FIC_index_) according to the following formulas [25,26]: FIC_index_ = FIC_OT_ + FIC_EO_; FIC = MIC_C_/MIC_I_.

According to [27] and the European Society of Clinical Microbiology and Infectious Diseases (ESCMID, Basel, Switzerland, 2023), a synergistic effect was considered when FIC_index_ ≤ 0.5; additive when 0.5 < FIC_index_ ≤ 1; indifferent when 1 < FIC_index_ < 2; and antagonism when FIC_index_ ≥ 2 [26].

## 3. Results

### 3.1. Susceptibility Test

The MIC_OT_ values obtained ranged from 256 to 512 µg/mL, confirming the resistance of all strains to this antimicrobial agent (≥16 µg/mL) [27]. The MIC_EO_ ranged from 312.5 to 1250 µg/mL, depending on the EO and the strain.

### 3.2. Antimicrobial Interaction Test

Table 3 shows the results of the interaction test of OT with the selected essential oils. A total of 80 OT and EO combinations were studied. We observed some variations in the MIC_I_ values when studying the products separately before the interaction test, which did not influence the results, since the calculation of the FIC and FIC_index_ was performed with the MIC_I_ and MIC_C_ obtained in the interaction test [26].

According to EUCAST guidelines (2000), a positive potentiation (FIC_index_ < 1) was observed between OT and the four EOs tested for at least one of the strains. This resulted in a reduction in the effective concentration of both products by 2 to 4 times their initial value in the case of oils and between 2 and 1000 times in the case of the antibiotic. No antagonistic effect was observed. The best results were obtained with cinnamon, since synergistic (FIC_index_ = 0.5) and additive effects (FIC_index_ = 0.625–1) were detected for all the strains. These effects were associated in three cases with a notable reduction (between 8 and 1024 times) in the effective concentration of OT: from 512 µg/mL to 0.5 µg/mL for the synergistic effect and to 64 µg/mL for the additive effect. In all these assays, the MIC of cinnamon was reduced by half, remaining between 39.062 and 625 µg/mL.

The combination of OT with clove and red thyme resulted in an additive effect in all cases (FIC_index_ = 0.625–1). Clove reduced the effective concentration of OT by 2–8 times and its own concentration by 2–4 times. Red thyme decreased the initial MIC of both products up to fourfold.

Finally, an additive effect was observed for oregano in four out of the five strains tested (FIC_index_ = 0.562–1), while the remaining strain showed indifference (FIC_index_ = 1.001). In the best assay (FIC_index_ = 0.562), the effective concentration of OT was reduced by 16-fold (from 512 µg/mL to 32 µg/mL) and that of oregano by two-fold (from 625 µg/mL to 312.5 µg/mL).

## 4. Discussion

Early research on the antimicrobial power against *Salmonella* of the active components present in the majority of the EOs included in this work [28] demonstrated that exposing multi-resistant strains of *S*. Typhimurium DT104, isolated from both pigs and humans, to sub-therapeutic doses of cinnamaldehyde (cinnamon EO) increased the sensitivity of both isolates to ampicillin, tetracycline, chloramphenicol, streptomycin, and sulfamethoxazole, making them susceptible to these antimicrobials. Thymol (oregano and red thyme EOs) increased the sensitivity of the bacteria to all antimicrobials tested except ampicillin. Carvacrol (oregano EO) increased the sensitivity of the human strain to chloramphenicol and sulfamethoxazole, and of the porcine strain to streptomycin and sulfamethoxazole.

Furthermore, Palaniappan and Holley [29] demonstrated a synergistic effect in the antimicrobial activity of tetracycline against *S*. Typhimurium when combined with cinnamaldehyde, carvacrol, and thymol (FIC_index_ = 0.1–0.37).

The precise mechanism by which natural antimicrobials reduce bacterial resistance to antibiotics is unknown. However, it is likely due to a structural change in the bacteria. Some studies suggest that these compounds may facilitate drug penetration through the outer layers of the cell wall, block the inhibitory effect of protective enzymes, or interfere with metabolic targets of the antibiotic [30,31,32]. Many authors suggest that whole EOs have greater antimicrobial potential than their single active ingredients due to the synergism between their molecules and the diversity of their mechanisms of action [25]. Another study conducted by Lauteri et al. [33] on tetracycline-resistant *Salmonella* strains demonstrated that combining this antibiotic with EOs from *Coridothymus capitatus* (olive thyme), *Eugenia caryophyllata* (clove), and *Thymus vulgaris* (common thyme) reduced the MIC of tetracycline from 256 to 4 μg/mL due to an increase in the sensitivity of all strains. However, the MIC of the EOs remained largely unchanged from their individual values, and in some cases, even increased.

In our study, the combination of OT with clove and red thyme only produced an additive effect, which contrasts with the results found elsewhere. Although the MIC_OT_ and MIC of EOs was reduced by 2 to 8-fold, the strains remained resistant to the antibiotic and the effective concentration of the EOs continued above the cytotoxicity threshold (500 μg/mL) [16]. However, it should be noted that the initial MIC of these EOs in our study (1250–5000 μg/mL and 625–1250 μg/mL for clove and red thyme, respectively) was significantly higher than that obtained by Lauteri et al. [33] for olive thyme, common thyme, and clove (<0.31–10 μg/mL, <0.31–5 μg/mL, and 0.31–20 μg/mL, respectively). This variation in antimicrobial activity may be attributed to differences in the origin, species, organ, and maturity of the plant, as well as the climatic and growing conditions, extraction method, and storage [34]. Some authors have reported differences in the activity of EOs, such as cinnamon, depending on the bacterial strain [34]. Previous work conducted to determine the in vitro susceptibility of the *Salmonella* strains included in our study revealed significantly (*p* < 0.05) higher susceptibility of *S.* Typhimurium to clove and *S*. Enteritidis to cinnamon. Additionally, *S.* Typhimurium isolates exhibited significantly higher MIC values for all the EOs tested (cinnamon, clove, oregano, red thyme, and common thyme). This indicates the presence of strains with reduced susceptibility to these compounds, which could explain the observed variability in the MIC of the AEs against the different strains in the present work [11].

Although cinnamon, which contains 69.8% cinnamaldehyde, showed the greatest potential to increase the antimicrobial activity of OT, it only managed to reduce its MIC to the sensitivity threshold (≤4 μg/mL) in one of the five strains tested (FIC_index_ = 0.5). However, this synergistic effect did not lead to a significant change in the MIC of cinnamon, which remained above the non-cytotoxic minimum concentration described by Fabio et al. [35] (0.05 μg/mL). The OT–oregano combination showed varying results, with a strong additive effect (FIC_index_ = 0.562) in some strains and no effect (FIC_index_ = 1.001) in others.

Essential oils, including oregano, cinnamon, and thyme, are commonly used in animal feed as feed additives and in the food industry for the development of new active packaging systems [36]. There is a paucity of genotoxicity studies of EOs and their components [37]. The results obtained by Llana-Ruiz-Cabello et al. [38] indicate that oregano essential oil (*Origanum vulgare*) does not have genotoxic effects in rats exposed to up to 200 mg/kg body weight (bw). Furthermore, the 90-day repeated-dose oral assay in rodents revealed no mortality or treatment-related adverse effects of the oregano EO in food/water consumption, body weight, hematology, biochemistry, necropsy, organ weight, and histopathology at a dose of 200 mg/kg body weight [39]. The limit value for lethal doses 50 (LD50) established by the OECD Test Guidelines for Chemicals is 2000 mg/kg (Organisation for Economic Co-operation and Development, 2008).

Previous studies have demonstrated that *Thymus vulgaris* essential oil has the potential to cause moderate acute oral toxicity in rats. After a single dose of 2000 mg/kg body weight, the lungs showed polymorphous nuclear infiltrates, hemosiderin macrophages, and thickening of the interstitial space [40]. In the repeated 28-day oral-dose toxicity studies conducted by the same authors, all rats treated with doses ≤ 250 mg/kg bw/day survived without organic, histopathological, and biochemical alterations. In the case of cinnamon essential oil (*Cinnamomum zeylanicum*), the in vitro cytotoxicity in the BSL (brine shrimp larvae) assay demonstrated a 50% lethal concentration (LC50 value of 0.03 μg/mL) [41].

With regard to in vivo tests, the EFSA expert panel recently conducted a study on the safety of oregano essential oil (*Origanum vulgare*) on various animal species (broilers, weaned piglets, and dairy cows), the consumer, the user, and the environment [42]. The results demonstrated that at the recommended use level (150 mg additive/kg feed), the product was safe for poultry and swine species reared for meat production. Additionally, doses of 500 mg additive/head/day (equivalent to ~25 mg/kg of complete feed) were also shown to be safe for dairy cows. The residue study demonstrated that the consumption of meat, liver, fatty milk, and eggs from these animals would not pose a safety concern for consumers. However, direct contact with the pure additive may cause skin and mucosal irritation and has the potential to cause sensitization in susceptible individuals. Its use in animal production is not expected to pose a risk to the environment [42].

## 5. Conclusions

Based on the study results, we consider combining EOs with OT to be an interesting alternative for controlling the development of *Salmonella* spp. strains resistant to this antibiotic. The results of this work enable a reduction in antibiotic use, thereby reducing the likelihood of creating new resistance and releasing antimicrobials into the environment. The synergies of essential oils and antimicrobials could be applied in both animals and humans, following the One Health approach. It is important to overcome limitations resulting from variations in chemical composition, expand in vitro studies to include more strains, determine the mechanisms of action of the combination of oxytetracycline and essential oils against multiresistant strains of *Salmonella enterica*, and conduct field tests to evaluate efficacy in animal models.

## Figures and Tables

**Table 1:** Description of *Salmonella enterica* strains used in this study.

Strain Ref.	Serotype	Phage Type	Origin	Related Pathology	Antibiogram
1	*S.* Typhimurium	204	Partridge	Digestive syndrome	A C S Su OT Cf
2	*S.* Typhimurium	U302	Swine	Septicaemia	A C S SxT OT Cf
3	*S.* Typhimurium	193	Partridge	Acute death	A C S SxT OT G Enr
4	*S.* London	-	Turkey	Carrier animal	A OT Cip Sxt Enr
5	*S.* Enteritidis	-	Laying hens	Carrier animal	OT Cip Nx Sxt Enr

A: ampicillin, C: chloramphenicol, S: streptomycin, Su: sulphonamide, SxT: trimetoprim-sulfamethoxazole, OT: tetracycline, Cf: cefalexin, G: gentamicin, Enr: enrofloxacina, Cip: ciprofloxacin, Nx: nalidixic acid.

**Table 2:** Botanical and chemical characteristics of the essential oils tested in this work.

Essential Oil	Common Name	Origin	Main Components
*Cinnamomum zeylanicum*	Cinnamon	Bark	Cinnamaldehyde (69.18%), linalool (3.19%), eugenol (3.03%)
*Eugenia caryophyllata*	Clove	Bud	Eugenol (85–90%), eugenyl acetate (5–10%), β-caryophyllene (0–5%)
*Origanum vulgare*	Oregano	Flowersand stems	Carvacrol (63.01%), thymol (10.56%), *γ*-terpinene (8.11%)
*Thymus zygis*	Red thyme	Air part	Thymol (46.9%), p-cymene (21.72%), *γ*-terpinene (9.32%), linalool (4.8%)

**Table 3:** Interaction assay of oxytetracycline (OT) and essential oils against multiresistant *Salmonella enterica* strains.

	*S.* Typhimurium 1			*S.* Typhimurium 2			*S.* Typhimurium 3			*S*. Enteritidis 5			*S.* London 4		
Interaction	MIC_I_	MIC_C_	FIC_index_ FIC	MIC_I_	MIC_C_	FIC_index_ FIC	MIC_I_	MIC_C_	FIC_index_ FIC	MIC_I_	MIC_C_	FIC_index_ FIC	MIC_I_	MIC_C_	FIC_index_ FIC
OT + Cin			0.625			0.5			1			0.625			0.625
OT	1024	512	0.5	512	0.5	0	512	256	0.5	512	64	0.125	512	64	0.125
Cinnamon	312.5	39.062	0.125	625	312.5	0.5	312.5	156.25	0.5	1250	625	0.5	1250	625	0.5
OT + Clove			0.625			0.75			1			1			0.625
OT	512	64	0.125	512	256	0.5	256	128	0.5	256	128	0.5	512	64	0.125
Clove	1250	625	0.5	1250	312.5	0.25	1250	625	0.5	2500	1250	0.5	2500	1250	0.5
OT + Ore			0.562			0.75			0.75			1.001			1
OT	512	32	0.062	512	128	0.25	512	128	0.25	256	0.5	0.001	256	128	0.5
Oregano	625	312.5	0.5	625	312.5	0.5	625	312.5	0.5	625	625	1	625	312.5	0.5
OT + Red th			1			1			0.75			0.75			1
OT	512	256	0.5	512	256	0.5	512	256	0.5	512	128	0.25	256	128	0.5
Red thyme	625	312.5	0.5	625	312.5	0.5	1250	312.5	0.25	625	312.5	0.5	1250	625	0.5

OT: oxytetracycline; Cin: cinnamon; Red th: red thyme; MIC_I_: individual minimum inhibitory concentration; MIC_C_: combined minimum inhibitory concentration; FIC: fractional inhibitory concentration; FIC_index_: fractional inhibitory concentration index.

## Data Availability

Data are contained within the article.

## References

[1] Hasan M., Wang J., Ahn J. (2023). Ciprofloxacin and Tetracycline Resistance Cause Collateral Sensitivity to Aminoglycosides in Salmonella Typhimurium. Antibiotics.

[2] Uddin T.M., Chakraborty A.J., Khusro A., Zidan B.R.M., Mitra S., Emran T.B., Dhama K., Ripon M.K.H., Gajdács M., Sahibzada M.U.K. (2021). Antibiotic resistance in microbes: History, mechanisms, therapeutic strategies and future prospects. J. Infect. Public Health.

[3] Dawan J., Ahn J. (2021). Assessment of cooperative antibiotic resistance of *Salmonella* Typhimurium within heterogeneous population. Microb. Pathog..

[4] EFSA Journal, 2023. The European Union Summary Report on Antimicrobial Resistance in Zoonotic and Indicator Bacteria from Humans, Animals and Food in 2020/2021. https://efsa.onlinelibrary.wiley.com/doi/10.2903/j.efsa.2023.7867#:~:text=Resistancewasfrequentlyfoundto,coliinsomecountries.

[5] Roope L.S.J., Smith R.D., Pouwels K.B., Buchanan J., Abel L., Eibich P., Butler C.C., Tan P.S., Sarah Walker A., Robotham J.V. (2019). The challenge of antimicrobial resistance: What economics can contribute. Science.

[6] Murugaiyan J., Anand Kumar P., Rao G.S., Iskandar K., Hawser S., Hays J.P., Mohsen Y., Adukkadukkam S., Awuah W.A., Jose R.A.M. (2022). Progress in Alternative Strategies to Combat Antimicrobial Resistance: Focus on Antibiotics. Antibiotics.

[7] Chouhan S., Sharma K., Guleria S. (2017). Antimicrobial Activity of Some Essential Oils—Present Status and Future Perspectives. Medicines.

[8] Fadli M., Chevalier J., Saad A., Mezrioui N.E., Hassani L., Pages J.M. (2011). Essential oils from Moroccan plants as potential chemosensitisers restoring antibiotic activity in resistant Gram-negative bacteria. Int. J. Antimicrob. Agents.

[9] Kon K.V., Rai M.K. (2012). Plant essential oils and their constituents in coping with multidrug-resistant bacteria. Expert Rev. Anti. Infect. Ther..

[10] Yap P.S.X., Yiap B.C., Ping H.C., Lim S.H.E. (2014). Essential Oils, A New Horizon in Combating Bacterial Antibiotic Resistance. Open Microbiol. J..

[11] Solarte A.L., Astorga R.J., Aguiar F., Galán-Relaño Á., Maldonado A., Huerta B. (2017). Combination of Antimicrobials and Essential Oils as an Alternative for the Control of *Salmonella enterica* Multiresistant Strains Related to Foodborne Disease. Foodborne Pathog. Dis..

[12] Burt S. (2004). Essential oils: Their antibacterial properties and potential applications in foods-A review. Int. J. Food Microbiol..

[13] Bakkali F., Averbeck S., Averbeck D., Idaomar M. (2008). Biological effects of essential oils-A review. Food Chem. Toxicol..

[14] Carson C.F., Hammer K.A., Thormar H. (2011). Chemistry and Bioactivity of Essential Oils. Lipids and Essential Oils as Antimicrobial Agents.

[15] Diaz-Sanchez S., D’Souza D., Biswas D., Hanning I. (2015). Botanical alternatives to antibiotics for use in organic poultry production. Poult. Sci..

[16] Dušan F., Marián S., Katarína D., Dobroslava B. (2006). Essential oils-their antimicrobial activity against *Escherichia coli* and effect on intestinal cell viability. Toxicol. Vitr..

[17] Penalver P., Huerta B., Borge C., Astorga R., Romero R., Perea A. (2005). Antimicrobial activity of five essential oils against origin strains of the *Enterobacteriaceae* family. Apmis.

[18] Solarte A.L., Astorga R.J., De Aguiar F.C., De Frutos C., Barrero-Domínguez B., Huerta B. (2018). Susceptibility Distribution to Essential Oils of *Salmonella enterica* Strains Involved in Animal and Public Health and Comparison of the Typhimurium and Enteritidis Serotypes. J. Med. Food.

[19] Nazzaro F., Fratianni F., De Martino L., Coppola R., De Feo V. (2013). Effect of essential oils on pathogenic bacteria. Pharmaceuticals.

[20] Langeveld W.T., Veldhuizen E.J.A., Burt S.A. (2014). Synergy between essential oil components and antibiotics: A review. Crit. Rev. Microbiol..

[21] Monte D.F.M., Tavares A.G., Albuquerque A.R., Sampaio F.C., Oliveira T.C.R.M., Franco O.L., Souza E.L., Magnani M. (2014). Tolerance response of multidrug-resistant *Salmonella enterica* strains to habituation to Origanum vulgare L. essential oil. Front. Microbiol..

[22] Lu F., Ding Y.C., Ye X.Q., Ding Y.T. (2011). Antibacterial effect of cinnamon oil combined with thyme or clove oil. Agric. Sci. China.

[23] (2024). Performance Standards for Antimicrobial Disk and Dilution Susceptibility Tests for Bacteria Isolated from Animals, 6th Edition.

[24] Si H., Hu J., Liu Z., Zeng Z.L. (2008). Antibacterial effect of oregano essential oil alone and in combination with antibiotics against extended-spectrum β-lactamase-producing *Escherichia coli*. FEMS Immunol. Med. Microbiol..

[25] Knezevic P., Aleksic V., Simin N., Svircev E., Petrovic A., Mimica-Dukic N. (2016). Antimicrobial activity of *Eucalyptus camaldulensis* essential oils and their interactions with conventional antimicrobial agents against multi-drug resistant *Acinetobacter baumannii*. J. Ethnopharmacol..

[26] Fratini F., Mancini S., Turchi B., Friscia E., Pistelli L., Giusti G., Cerri D. (2017). A novel interpretation of the Fractional Inhibitory Concentration Index: The case *Origanum vulgare* L. and *Leptospermum scoparium* J. R. et G. Forst essential oils against *Staphylococcus aureus* strains. Microbiol. Res..

[27] (2000). EUCAST Terminology relating to methods for the determination of susceptibility of bacteria to antimicrobial agents. Clin. Microbiol. Infect..

[28] Johny A.K., Hoagland T., Venkitanarayanan K. (2010). Effect of subinhibitory concentrations of plant-derived molecules in increasing the sensitivity of multidrug-resistant *Salmonella enterica* serovar Typhimurium DT104 to antibiotics. Foodborne Pathog. Dis..

[29] Palaniappan K., Holley R.A. (2010). Use of natural antimicrobials to increase antibiotic susceptibility of drug resistant bacteria. Int. J. Food Microbiol..

[30] Darwish R.M., Aburjai T., Al-Khalil S., Mahafzah A. (2002). Screening of antibiotic resistant inhibitors from local plant materials against two different strains of Staphylococcus aureus. J. Ethnopharmacol..

[31] Aburjai T., Darwish R.M., Al-Khalil S., Mahafzah A., Al-Abbadi A. (2001). Screening of antibiotic resistant inhibitors from local plant materials against two different strains of *Pseudomonas aeruginosa*. J. Ethnopharmacol..

[32] Hemaiswarya S., Kruthiventi A.K., Doble M. (2008). Synergism between natural products and antibiotics against infectious diseases. Phytomedicine.

[33] Lauteri C., Maggio F., Serio A., Festino A.R., Paparella A., Vergara A. (2022). Overcoming Multidrug Resistance in *Salmonella* spp. Isolates Obtained from the Swine Food Chain by Using Essential Oils: An in vitro Study. Front. Microbiol..

[34] Santoyo S., Cavero S., Jaime L., Ibañez E., Señoráns F.J., Reglero G. (2006). Supercritical carbon dioxide extraction of compounds with antimicrobial activity from *Origanum vulgare* L.: Determination of optimal extraction parameters. J. Food Prot..

[35] Fabio A., Cermelli C., Fabio G., Nicoletti P., Quaglio P. (2007). Screening of the antibacterial effects of a variety of essential oils on microorganisms responsible for respiratory infections. Phyther. Res..

[36] Radha Krishnan K., Babuskin S., Azhagu Saravana Babu P., Sasikala M., Sabina K., Archana G., Sivarajan M., Sukumar M. (2014). Antimicrobial and antioxidant effects of spice extracts on the shelf life extension of raw chicken meat. Int. J. Food Microbiol..

[37] Slameňová D., Horváthová E., Wsólová L., Šramková M., Navarová J. (2009). Investigation of anti-oxidative, cytotoxic, DNA-damaging and DNA-protective effects of plant volatiles eugenol and borneol in human-derived HepG2, Caco-2 and VH10 cell lines. Mutat. Res. Genet. Toxicol. Environ. Mutagen..

[38] Llana-Ruiz-Cabello M., Maisanaba S., Puerto M., Prieto A.I., Pichardo S., Moyano R., González-Pérez J.A., Cameán A.M. (2016). Genotoxicity evaluation of carvacrol in rats using a combined micronucleus and comet assay. Food Chem. Toxicol..

[39] Llana-Ruiz-Cabello M., Maisanaba S., Puerto M., Pichardo S., Jos A., Moyano R., Cameán A.M. (2017). A subchronic 90-day oral toxicity study of *Origanum vulgare* essential oil in rats. Food Chem. Toxicol..

[40] Rojas-Armas J., Arroyo-Acevedo J., Ortiz-Sánchez M., Palomino-Pacheco M., Castro-Luna A., Ramos-Cevallos N., Justil-Guerrero H., Hilario-Vargas J., Herrera-Calderón O. (2019). Acute and repeated 28-day oral dose toxicity studies of *Thymus vulgaris* L. essential oil in rats. Toxicol. Res..

[41] Sharififar F., Moshafi M.H., Dehghan-Nudehe G., Ameri A., Alishahi F., Pourhemati A. (2009). Bioassay screening of the essential oil and various extracts from 4 spices medicinal plants. Pak. J. Pharm. Sci..

[42] Bampidis V., Azimonti G., Bastos M.D.L., Christensen H., Dusemund B., Kouba M., Kos Durjava M., López-Alonso M., López Puente S., Marcon F. (2019). Safety of an essential oil from *Origanum vulgare* subsp. *hirtum* letsw. var. Vulkan when used as a sensory additive in feed for all animal species. EFSA J..

